# National consensus statement by the Austrian Societies for Rheumatology, Pulmonology, Infectiology, Dermatology and Gastroenterology regarding the management of latent tuberculosis and the associated utilization of biologic and targeted synthetic disease modifying antirheumatic drugs (DMARDs)

**DOI:** 10.1007/s00508-022-02062-7

**Published:** 2022-08-29

**Authors:** Eva Rath, Michael Bonelli, Christina Duftner, Johann Gruber, Peter Mandl, Florentine Moazedi-Furst, Herwig Pieringer, Rudolf Puchner, Holger Flick, Helmut J. F. Salzer, Günter Weiss, Stefan Winkler, Hans Skvara, Alexander Moschen, Harald Hofer, Julia Feurstein, Judith Sautner

**Affiliations:** 1 Austrian Society for Rheumatology and Rehabilitation (ÖGR), Vienna, Austria; 2 Austrian Society for Pulmonology (ÖGP), Vienna, Austria; 3 Austrian Society for Infectiology (ÖGIT), Kottingbrunn, Austria; 4 Austrian Society for Dermatology and Venerology (ÖGDV), Vienna, Austria; 5 Austrian Society for Gastroenterology and Hepatology (ÖGGH), Vienna, Austria; 6 1. Medical Department, Hanusch Hospital, Vienna, Austria; 7 Department of Medicine III, rheumatology, Medical University of Vienna, Vienna, Austria; 8 Department of Medicine II, Medical University of Innsbruck, Innsbruck, Austria; 9 Department of Rheumatology and Immunology, Medical University of Graz, Graz, Austria; 10 Diakonissen Hospital, Linz, Austria; 11 Private practice, Wels, Austria; 12 Department of Pulmonology, Medical University of Graz, Graz, Austria; 13 Department of Pulmonology, Kepler Medical University, Linz, Austria; 14 Department of Infectiology and Tropical Diseases, Medical University of Vienna, Vienna, Austria; 15 Department of Dermatology, State Hospital Wiener Neustadt, Wiener Neustadt, Austria; 16 Department of Gastroenterology and Hepatology, Kepler Medical University, Linz, Austria; 17 Department of Medicine 1, Wels-Grieskirchen Clinics, Wels, Austria; 18 Department of Medicine II, Lower Austrian Centre for Rheumatology, Karl Landsteiner Institute for Clinical Rheumatology, State Hospital Stockerau, Landstr. 18, 2000 Stockerau, Austria; 19 Medical University of Vienna, Vienna, Austria

**Keywords:** Tuberculosis, Latent tuberculosis, Incidence, Biologic and targeted synthetic DMARDs, Antibiotic regimen

## Abstract

This publication provides a thorough analysis of the most relevant topics concerning the management of latent tuberculosis when using biologic and targeted synthetic Disease Modifying Antirheumatic Drugs (DMARDs) by a multidisciplinary, select committee of Austrian physicians. The committee includes members of the Austrian Societies for Rheumatology and Rehabilitation, Pulmonology, Infectiology, Dermatology and Gastroenterology. Consensus was reached on issues regarding screening and treatment of latent tuberculosis and includes separate recommendations for each biologic and targeted synthetic DMARD.

## Introduction

In 2011 the first Austrian consensus on the handling of latent tuberculosis ahead of initiating a treatment with biologic disease modifying antirheumatic drugs (bDMARDs) was established [1]. While relevant new insights into the safety of specific medications were achieved during the last decade, in addition many new products were introduced. Therefore, an expert group consisting of members of the Austrian Societies for Rheumatology and Rehabilitation (ÖGR), Pulmonology (ÖGP), Gastroenterology and Hepatology (ÖGGH), Dermatology and Venerology (ÖGDV), and Infectiology (ÖGIT) decided to develop recommendations for the distinct diagnosis as well as the management of latent tuberculosis before the start of a biologic (b) or targeted synthetic (ts) DMARD treatment and summarized their consensus hereinafter.

### Incidence of active tuberculosis and latent tuberculosis

Despite a worldwide decline of cases, tuberculosis is still an immense global health problem today. According to a WHO report, 10 million people contracted tuberculosis in 2019, and 1.4 million people even died subsequently [2]. However, the incidence of this infectious disease is unequally distributed worldwide, leading to a considerable variability when comparing different countries. Nearly half of the affected patients are living in only 30 countries, all of them having a poor national socioeconomic care in common.

**Fig. 1:**
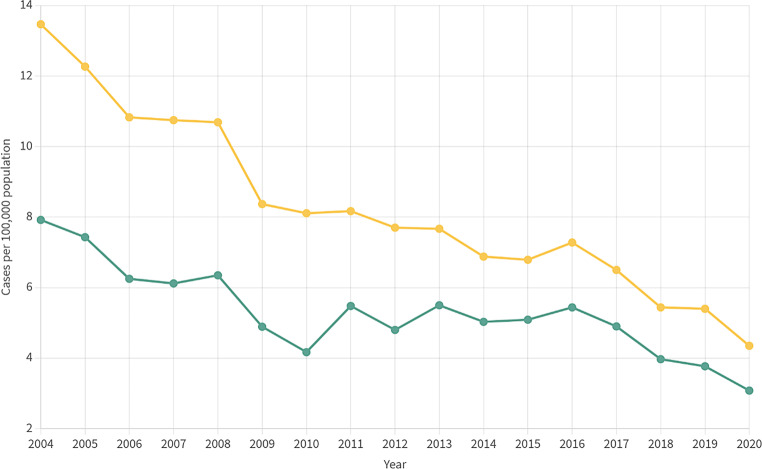
Reported cases of tuberculosis (*yellow line*) and microbiologically confirmed cases of *Mycobacterium tuberculosis* complex (*green line*) per 100,000 population in Austria (source: [46])

In comparison, Austria, like most north, central and western European countries, is showing a very low tuberculosis incidence: 4.4 cases per 100,000 inhabitants, and 388 cases nationwide in the year 2020. The incidence has further decreased compared to 2011 ([3]; Fig. 1).

In contrast to the distribution of manifest tuberculosis, the prevalence of latent tuberculosis is still unclear. Latent tuberculosis (LTBI) is defined as the asymptomatic persistence of vital tuberculous mycobacteria in the organism following an infection. The infected person is clinically healthy and not contagious. If a treatment such as immunosuppressants is given, the steady state between immunologic control and bacterial activity may be shifted to the disadvantage of immunity, and LTBI can progress to active tuberculosis, representing a reactivation. A positive interferon gamma release assay (IGRA) as well as a tuberculin skin test (TST) are proof of prior immunologic response to the pathogen. Given that the clinical examination and the chest x‑ray are without pathological findings, active tuberculosis is excluded though [4].

### Mode of action of various b-DMARDs and ts-DMARDs, potential influence on a tuberculosis infection and associated recommendations in the product information of medications (order on the basis of specializations and the date of product placement)

#### Anti-CD20-antibody, rituximab

This antibody directed against B cells, has been used in the treatment of lymphomas since 1998. In 2006, it received approval for the treatment of rheumatoid arthritis (RA). Since then, a large number of randomized, controlled trials, along with observational studies and registry data, have shown no evidence of an increased incidence of tuberculosis [5]. Even in countries showing a high incidence of tuberculosis, no safety risk was identified in this context [6]. The European Society of Clinical Microbiology and Infectious Diseases (ESCMID) issued a clear statement on this issue in 2018, which underlined the safety of this antibody with regard to tuberculosis [7]. In addition, no warning on this matter can be found in the drug’s technical product summary.

#### Tumor Necrosis Factor alpha (TNF) inhibitors (blockers)

When the first TNF blocker (infliximab) was released for prescription around the turn of the Millennium, an increased occurrence of tuberculosis reactivation soon became apparent during ongoing treatment. This subsequently led to routine screening for latent tuberculosis before commencing treatment [8]. Analysis of various registry data showed that the risk of contracting active tuberculosis is increased about fourfold during therapy [9]. However, there were specific variations in the incidence of tuberculosis reactivation between the individual TNF blockers. There were significantly fewer tuberculosis cases reported when the fusion protein etanercept was prescribed, and thus it likely carries a lower risk than the others [10, 11].

Due to lessons learned during the market launch phase of the TNF blockers, the biologic therapies studied thereafter were applied only after LTBI had been excluded or treated. Since that time, the testing and treatment of LTBI has been recommended in the summary of product characteristics provided by the manufacturers, even for substances which, from a physiological point of view, have no significant influence on the immune response against mycobacterial infection and also show no indications of an increased risk of tuberculosis, according to studies.

#### Interleukin(IL)-1 blockers, anakinra, canakinumab

The first antibody against IL‑1, a central element of the innate immune defence system, was introduced in 2002. The significance of this cytokine in defending against mycobacteria is not entirely clear [12]. However, in registry studies as well as during further observation, there was never cause to suspect an increased occurrence of tuberculosis [13]. In addition, although most of the studies on RA were conducted mainly in countries with a low incidence of tuberculosis, no cases of tuberculosis occurred in studies on the treatment of Behcet’s disease either [14].

Nonetheless, screening for LTBI is recommended for inclusion in the summary of product characteristics for anakinra and canakinumab.

#### Anti-CD80/86, abatacept

Upon discovery of cases of tuberculosis coinciding with the market launch of the TNF blockers, screening for LTBI was mostly carried out in the approval and dose-finding studies around the T‑cell co-stimulation inhibitor abatacept. However, in the more than 15 years since market launch (2005 in the USA and 2007 in Europe), there has been no reported evidence of an increased incidence of tuberculosis. Numerous registry data and observational studies (some from countries reporting higher incidences of tuberculosis) showed no or only very isolated cases of tuberculosis during therapy with abatacept [6]. In 2018, an analysis of data from several large abatacept trials totalling 21,335 patient-years found only 17 cases of tuberculosis, all in high-risk countries [13, 15]. These data thus indicate an extremely low risk of tuberculosis infection when prescribing abatacept. Nevertheless, screening for LTBI is still recommended for inclusion in the summary of product characteristics.

#### Anti-Interleukin (IL) 6 receptor antibody, tocilizumab, sarilumab

With tocilizumab (IL‑6 receptor blockade), doctors wisely decided to proceed with great caution after noting the results reported after prescribing TNF blockers. Administration of the drug during approval studies was only carried out after the exclusion of LTBI, with the result that the actual risk of tuberculosis associated with this therapy remained unexplored. Registry data, observational studies as well as individual reports of untreated LTBI under tocilizumab did not indicate a tuberculosis risk associated with this class of drugs, but since routine screening before treatment was recommended, the overall assessment favored preventive tuberculosis treatment [6, 12, 13, 15]. The summary of product characteristics also recommends screening for and treatment of LTBI.

#### Anti-Interleukin(IL)-12/23 antibody, ustekinumab

Inhibiting IL-12 and IL-23 theoretically leads to an impairment of the immune response against mycobacteria [16]. Observations since market launch in 2009 have not shown an increased incidence of tuberculosis cases connected with ustekinumab treatment so far [6, 12, 15]. However, as with the aforementioned medications, LTBI was screened for in studies and also prior to widespread use before commencing treatment, which adds to the difficulty of making any final assessment. Various reviews have assessed the risk of tuberculosis activation as very low. The screening and treatment of LTBI is clearly recommended in the summary of product characteristics supplied with ustekinumab.

#### Anti-B lymphocyte Stimulator (BLyS), belimumab

This antibody treatment, directed against the B‑lymphocyte stimulating factor (BLyS), leads to a lifespan and activity reduction of B‑lymphocytes. This therapy has been approved for the treatment of systemic lupus erythematosus (SLE) since 2011. There is neither a suspected risk of tuberculosis, nor have studies shown the occurrence of tuberculosis cases [17]. In most studies, the words mycobacteria and tuberculosis are not even mentioned. According to the summary of product characteristics for belimumab, any risk associated with latent or active tuberculosis remains unknown.

#### Phosphodiesterase 4 (PDE4) inhibitor, apremilast

The phosphodiesterase 4 (PDE4) inhibitor was the first drug to be included in the group of targeted synthetic (tsDMARDs), which exert their effect by influencing signalling pathways within the cells. Apremilast, which came onto the market in 2015, leads to an increase in cyclic adenosine monophosphate (cAMP) through inhibition of PDE4 and thus to reduced formation and release of inflammatory mediators. Interestingly, there is actually no clinically relevant impairment of the immune response with regard to infections when the drug is used, and it is also considered safe in LTBI [6, 18]. There is also no mention of any concerns regarding tuberculosis infection in the summary of product characteristics.

#### Anti-Interleukin(IL)-17, secukinumab, ixekizumab, brodalumab

The first representative of this drug class also came onto the market in the same year as apremilast. With the inhibition of IL-17, the effect is aimed in particular at T helper (Th) 17 cells. Any relevant influence regarding infections seems to be only for the control of *Candida*. There are no indications of a reduced mycobacterial immune response [12, 19].

There is also no evidence of an increased risk of tuberculosis infection with anti-IL-17 in neither studies from registry data nor from observational studies [6, 15, 18]. In addition, there are case reports and case series, where patients with LTBI received anti-IL-17 therapy without preventive therapy and not a single case of tuberculosis infection occurred [20].

However, in the approval studies of IL-17 blockers, LTBI was always screened for and treated as necessary, so that no evidence-based statement can be made on the definitive risk of tuberculosis in this context.

In light of numerous indirect indications of harmlessness with regard to the risk of tuberculosis, the summary of product characteristics of the various IL-17 blockers only suggests that screening can be considered or contemplated.

#### Anti-Interleukin (IL) 23, guselkumab, risankizumab, tildrakizumab

A treatment to inhibit anti-IL-23 has been approved since 2017. Inhibiting this cytokine influences the activity of various cells of the innate and adaptive immune systems, in particular T cells, macrophages and dendritic cells, and thus theoretically also has an influence on the immune response against mycobacteria [21]. In registered trials for anti-IL 23 therapies, LTBI was always screened for and, if present, mostly treated. To date, no reactivation of tuberculosis has occurred in either clinical or real-world studies [22].

The product information for anti-IL-23 medications states that LTBI should be screened for and treatment should be considered.

#### Janus kinase (JAK) inhibitor, tofacitinib, baricitinib, upadacitinib, filgotinib

The first representative of the JAK inhibitors, tofacitinib, has been approved since 2017 and thus expanded the group of tsDMARDs. Inhibiting Janus kinases, which are relevant for the signalling effect of various cytokines from the cell surface into the cell nucleus, results in the immunomodulatory anti-inflammatory effect. The influence on the immune response against mycobacteria is estimated to be similar to that of TNF blockers [6, 23–25]. However, there are no data on this matter because from the beginning their use only occurred after exclusion or treatment of LTBI. In the extended observation period of the phase II and III trials of tofacitinib, 26 tuberculosis infections were found in 5671 patients located mainly in high-risk environments, suggesting a rather low risk of infection and reactivation [23]. Technical information available for the various JAK inhibitors is worded in different ways. Screening should be done in all cases. Regarding preventive treatment, recommendations range from “should be considered” (baricitinib, upadacitinib) to “LTBI should be treated” (tofacitinib, filgotinib).

#### Receptor activator of NF-κB ligand (RANKL) inhibitor, denosumab

RANKL is responsible for the conversion of precursor cells into bone-degrading osteoclasts; its inhibition thus reduces bone resorption. Any additional impact on the immune system is unknown, which is why relevance in the mycobacterial immune response is not assumed. The drug has been approved since 2010 and is widely used in the treatment of osteoporosis. The drug appears to be harmless with regard to tuberculosis, and tuberculosis is not mentioned in the summary of product characteristics [26]. There is even one case report of successful therapeutic use of denosumab during active tuberculosis with hypercalcemia [27].

#### Sclerostin inhibitor, romosozumab

The antibody against sclerostin, European Medicines Agency (EMA) approval in 2019, has an isolated influence on bone formation but shows no additional immunosuppressive effect. Influence on tuberculosis infection is not assumed and is therefore not mentioned in the product summary [28].

#### Integrin blocker, vedolizumab

The integrin blocker vedolizumab, approved in 2014, prevents the docking of activated lymphocytes in the intestinal tissue [29]. Despite this intestinal-specific effect and no reports of tuberculosis infections of patients undergoing treatment, the drug’s summary of product characteristics formulates that LTBI must be examined and treated if necessary.

#### Anti-Immunglobuline (Ig) E, omalizumab

The antibody against IgE has been approved since 2005 and is used to treat allergic asthma, chronic rhinosinusitis with polyps and chronic spontaneous urticaria. Neither theoretically nor in observational studies is an increased risk of tuberculosis apparent [12]. There is also no reference made in the product summary.

#### Anti-Complement (C) 5(a), eculizumab, ravulizumab, avacopan

Antibodies that block a protein of the terminal activation pathway of the complement are associated with a susceptibility to meningococcal infections (eculizumab, ravulizumab). Avacopan an antibody against the receptor of C5a carries no such risk [12, 30]. With regard to mycobacterial infections, there are no indications of increased risk, nor is there a mention in the product summary.

#### Anti-Interleukin(IL)-5, mepolizumab, reslizumab

Antibodies against IL‑5 are used to treat severe eosinophilic asthma and have no expected effect on the mycobacterial immune response. Studies have also shown no evidence in this regard [12]. The technical product summary also makes no mention of tuberculosis.

#### Anti-Interleukin(IL)-4R/Anti-Interleukin(IL)-13R, dupilumab

Influence on the immune response against mycobacteria has neither come to light nor observed for the antibody against IL-4R and IL-13R [31]. There is also no indication of this in the product summary.

#### Anti-Interferon alpha beta receptor(IFNAR)1, anifrolumab

This human antibody to the type I interferon receptor subunit 1 inhibits signalling by all type 1 interferons and has been approved for the treatment of moderate to severe SLE by EMA in 2022. Interferon alpha seems to play a role in the cellular response to mycobacteria infection. Since it is involved in the balance between host defense and inflammatory reactions, the effect of blocking its function is not totally clear yet [32–34]. In the phase II and III studies on anifrolumab, LTBI was an exclusion criterion, like in other studies assessing the efficacy of biologicals. In the pooled data of the TULIP I and II studies, 4 cases of LTBI (IGRA turned positive without radiographic or clinical signs of tuberculosis) occurred in 459 patients receiving anifrolumab, but no case of active tuberculosis was observed [35].

The product summary advises to consider preventive tuberculosis treatment in case of untreated LTBI before starting anifrolumab.

### Existing international recommendations on LTBI and b-DMARDs/ts-DMARDs

Guidelines for the diagnosis and therapy of LTBI differ only insignificantly among each other [36–38]. For the diagnosis of LTBI, an IGRA and/or TST is always recommended. There are 4 therapeutic regimens available for the treatment of LTBI: isoniazid (INH) for 6–9 months, rifampicin (RIF) for 3–4 months, INH together with RIF for 3–4 months or rifapentine with INH weekly for 3 months. The dosage in each case is given as 5 mg/kg body weight for INH (maximum 300 mg/day), or 10 mg/kg body weight for RIF (maximum 600 mg/day). The weighting of the different regimens is slightly different in the three publications mentioned, but overall these therapies are considered equivalent. Rifapentine is not available in Austria and is therefore not administered.

Regarding the management of LTBI in the context of b‑DMARD/ts-DMARD treatment, there are no clear recommendations in international medical society publications. In the current American College of Rheumatology (ACR) guidelines for the treatment of RA, published in 2021, a reference can be found for abatacept to be used before other b‑DMARD/ts-DMARDs for non-tuberculous mycobacterial infections [39]. The 2015 ACR guidelines recommend screening with IGRA or TST and, if appropriate, preventive tuberculosis therapy before initiating biologics or tofacitinib [40]. Recent recommendations on the management of RA from the European Alliance of Associations for Rheumatology (EULAR) do not specifically address the treatment of LTBI [41]. In the 2013 guidelines, rituximab therapy is recommended for LTBI and contraindications to chemoprophylaxis [42]. The British Society of Rheumatology (BSR) published “biologic DMARD safety guidelines in inflammatory arthritis” in 2019 [43]. Chemoprophylaxis is recommended prior to biologic treatment. However, it is emphasized that the probability of tuberculosis reactivation under rituximab and abatacept appears to be quite low. For screening, the BSR guidelines recommend a combination of chest X‑ray and IGRA or TST. In the German consensus based, following a structured process (S2K) guidelines on tuberculosis in adults from 2017, a chapter is dedicated to LTBI with TNF inhibitors and other biologics [38]. This refers to the SAFEBIO study [13], where a low to no risk for activating tuberculosis was found for rituximab, abatacept, tocilizumab, ustekinumab and anakinra. IGRA and/or TST are recommended as screening methods. The German S3 (containing all elements of systematic guideline development) guidelines on psoriasis from 2021 include a separate chapter on dealing with LTBI. Indeed, they recommend screening for LTBI before bDMARD therapy (anti-TNF, anti-IL17, anti-IL12/23, anti-IL-23) and preventive treatment of LTBI, but emphasize that there is no known risk of reactivation [44].

## Methods

The Select Committee represents a broad cross-section of the Austrian rheumatological profession with 8 rheumatologists (university, non-university, private practice, members of the board of the Austrian Society for Rheumatology and Rehabilitation) as well as 2 infectiologists, 2 pulmonologists (working group leader for infectious diseases and tuberculosis of the Austrian Society for Pulmonology, ÖGP), one dermatologist (working group leader for biologics and immunotherapy of the Austrian Society for Dermatology and Venerology, ÖGDV) and one gastroenterologist (working group leader for chronic inflammatory bowel diseases of the Austrian Society for Gastroenterology and Hepatology, ÖGGH). In addition, writing, editing and organizational support was provided (JF), as well as advisory support regarding hepatological issues (HH).

After conducting an extensive literature search on the incidence, occurrence and treatment for tuberculosis under various b/ts-DMARDS as well as reviewing guidelines from other countries and cross-referencing technical information for each of the drugs approved in Austria, a key member (ER) forwarded the compiled information to all consensus participants and 8 salient issues were formulated as questions.

During an initial (virtual) consensus meeting of the committee on 19 April 2021, the topic was discussed in detail and unanimous agreement was reached on the relevant issues.

At a further (virtual) meeting with eight committee members, open issues were discussed. Individual meetings and individual correspondence with committee members who were unable to attend this meeting were then held to share the resolutions discussed in the group meeting.

Subsequently, a total of 37 statements were formulated and sent to all members. A vote was cast for each point on a Likert scale of 1–5 (strongly agree, agree, neither agree nor disagree, disagree, strongly disagree). A > 75% consensus (strongly agree or agree) was achieved on all points. After the results were shared with all participants (modified Delphi technique), there was further discussion, partly via text and partly oral, and a further round of voting, which then resulted in the final tallies.

After the statements were recorded in this article, the full document was sent out to all participants for correction and finally submitted for publication after processing comments and getting approval by all members and their professional affiliates.

## Consensus findings

### When should be screened for latent tuberculosis?

**Fig. 2:**
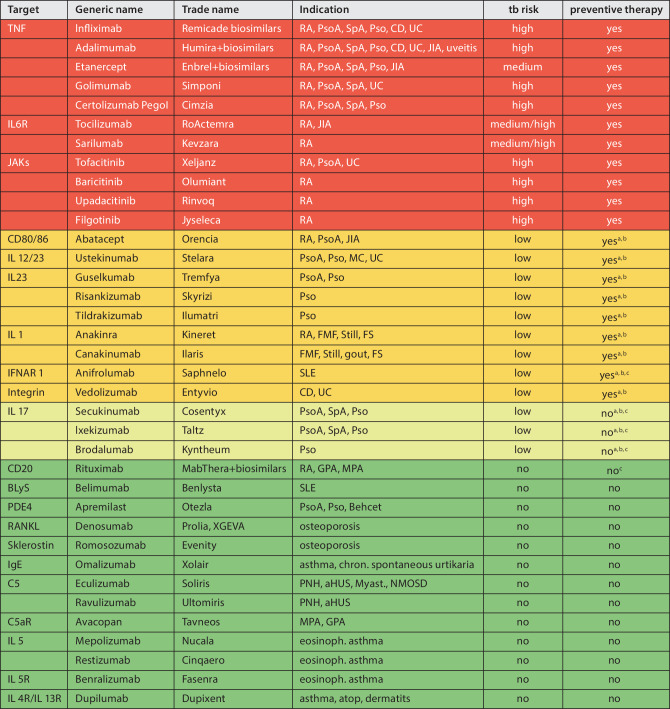
Heat map of bDMARDs and tsDMARDS regarding the risk of tuberculosis and need for preventive treatment; *red*: high risk, preventive treatment necessary, *orange*: low risk, preventive treatment necessary, *yellow*: low risk, preventive treatment not necessary, *green*: no risk, preventive not necessary. *TNF* tumor necrosis factor, *IL* interleukin, *IFNAR1* type I interferon receptor, *JAKs* Janus kinase inhibitor, *BlyS* B-lymphocyte stimulator, *PDE4* phosphodiesterase 4 inhibitor, *RANKL* receptor activator of NF-κB ligand, *IgE* immunoglobulin E, *RA* rheumatoid arthritis, *PsoA* psoriatic arthritis, *SpA* spondylarthritis, *Pso* psoriasis, *CD* Crohn’s disease, *UC* ulcerative colitis, *JIA* juvenile idiopathic arthritis, *FMF* familial Mediterranean fever, *Still* systemic juvenile idiopathic arthritis, adult onset Still’s disease, *FS* fever syndromes, *SLE* systemic lupus erythematosus, *PNH* paroxysmal nocturnal hemoglobinuria, *aHUS* atypical hemolytic uremic syndrome, *Myast.* myasthenia gravis, *NMOSD* neuromyelitis optica spectrum disorder, *MPA* microscopic polyangiitis, *GPA* granulomatosis with polyangiitis. ^a^Low incidence, due to routinely screening in studies. ^b^Theoretical risk low. ^c^According to the summary of product characteristics, this does not require screening

- Before starting bDMARD or tsDMARD therapy requiring preventive TB therapy, LTBI must be investigated/screened for (see heat map in Fig. 2: red, orange): 100% consensus.

The decision to screen for LTBI depends on which therapy has been selected. Those drugs that do not carry an increased risk for reactivation of LTBI do not require screening. Due to the occurrence of tuberculosis cases in the early years of TNF blocker treatments, physicians were sensitized to this issue, which is why LTBI often became a reason for exclusion in approval studies for newer bDMARDs and tsDMARDs or LTBI was treated preventively. For this reason, there is a lack of valid data on the true risk of tuberculosis reactivation for most therapies, so that the assessment by the panel of experts was based on the existing published case series, national registry data, post-marketing surveillance and the physiological significance of the respective drug with regard to mycobacterial immune response. Based on available data, a risk assessment was carried out, which is shown in Fig. 2 (heat map). In this instance, risk classification was carried out, on the one hand due to the theoretical pathophysiological influence of the medication and on the other hand on the basis of available data regarding the occurrence of tuberculosis while undergoing the various treatments. In addition, technical product summaries, some of which absolutely demand tuberculosis screening, were included in the respective classification. The classification was marked red (high risk, preventive treatment necessary), orange (low risk, preventive treatment necessary), yellow (low risk, preventive treatment not necessary), and green (no risk, preventive treatment not necessary).

### Ahead of which bDMARDs/tsDMARDs should be treated for latent tuberculosis?

Preventive tuberculosis therapy is indicated for the following drugs (heat map red and orange):

- Anti-TNF: 100% agreement
- Anti-IL6: 100% agreement
- JAK inhibitors: 100% agreement
- Abatacept: 100% agreement
- Anti-IL12/23: 100% agreement
- Anti-IL23: 80% agreement
- Anti-IL1: 80% agreement
- Anti-IFNAR1: 100% agreement
- Vedolizumab: 66% agreement

Preventive tuberculosis therapy is NOT indicated for the following drugs (heat map yellow and green):

- Anti-IL17: 86% agreement
- Anti-CD20: 100% agreement
- Anti-BLyS: 100% agreement
- Apremilast: 100% agreement
- RANKL inhibitor: 100% agreement
- Sclerostin inhibitor: 100% agreement
- Anti-IgE: 93% agreement
- Anti-C5: 93% agreement
- Anti-IL5: 93% agreement
- Anti-IL4: 93% agreement

The international guidelines for the treatment of rheumatological diseases with bDMARDs/tsDMARDs do not provide any clear recommendations regarding preventive tuberculosis therapy. The issue is either not addressed or formulated in very general terms and left to the decision of the practitioner based on individual risk-benefit analysis.

Existing publications on all bDMARD/csDMARD therapies were screened and assessed with regard to tuberculosis risk, and each member voted on the necessity of preventive tuberculosis therapy for each individual bDMARD and tsDMARD. Except for three groups of drugs, there was unanimous agreement regarding risk assessment and the need for preventive treatment (see Results).

For anti-IL‑1, two participants were against the implementation of preventive treatment and one participant was undecided. The decision in favor of preventive treatment was mainly based on the product summaries for anakinra and canakinumab, where testing for latent tuberculosis is recommended.

For anti-IL-23 therapy, there was one vote against the implementation of preventive tuberculosis treatment and two undecided participants. There is little theoretical influence on the mycobacterial immune response with this drug class and also no reports of tuberculosis reactivation in the literature. However, as the drug information also recommends the investigation of LTBI here, the majority vote was in favor of preventive tuberculosis treatment.

The decision was different for anti-IL-17. Here, only one participant was in favor of carrying out preventive treatment and one participant was undecided. With this class of drugs, too, the lack of influence with regard to mycobacterial immune response and the lack of reports of tuberculosis reactivation raised no objections to prescribing these medicines without preventive treatment. In addition, examining the product summaries screening for LTBI is not necessarily required for this class of drugs, but only to be taken into consideration. These factors ultimately led to the clear voting result against preventive treatment. Of course, the practitioner can still carry out an IGRA test and, if the result is positive, inform the patient about a theoretically low risk that cannot be completely ruled out.

In the case of vedolizumab, a drug that is only used in gastroenterology, three participants were undecided and two against the implementation of preventive treatment. Since the gastroenterological representative was clearly in favor of treatment which is quite clearly recommended in the product summary, the recommendation in favour of treatment was thus made, despite only 66% agreement.

Table 1 shows the summary of the facts leading to the panel decisions.

**Table 1:** Summary of facts leading to panel decisions

Target	TB testing necessary panel decision (%)	Theoretical TB risk	Evidence for TB risk	Product summary sheet clearly recommends testing
*TNF*	100	Yes	Yes (8, 9, 10, 11)	Yes
*IL 6R*	100	Yes?	No (6, 12, 13, 15)	Yes
*JAKi*	100	Yes?	No (6, 23, 24, 25)	Yes
*CD80/86*	100	No?	No (6, 13, 15)	Yes
*IL 12/23*	100	Yes?	No (6, 12, 15)	Yes
*IL 23*	80	Yes?	No (21, 22)	Yes
*IL 1*	80	Not clear	No (13, 14)	Yes
*IFNAR1*	100	Not clear	No (32, 33, 34, 35)	No
*Integrin*	66	No	No (29)	Yes
*IL 17*	7	No	No (6, 15, 18, 20)	No
*CD20*	0	No	No (5, 6, 7)	No
*BLyS*	0	No	No (17)	No
*PDE4*	0	Yes?	No (6, 18)	No
*RANKL*	0	No	No (26, 27)	No
*Sklerostin*	0	No	No (28)	No
*IgE*	0	No	No (12)	No
*C5*	0	No	No (12)	No
*IL 5*	0	No	No (12)	No
*IL 4R/13R*	0	No	No (31)	No

References providing evidence for TB risk have been put in brackets according to their number in the reference list.

*TNF* tumor necrosis factor, *IL 1R* Interleukin 1 receptor, *JAKi* januskinase inhibitor, *CD80/86* abatacept, *IL 12/23* Interleukin 12/23, *IL23* Interleukin 23, *IL 1* Interleukin 1, *IFNAR1* Anti Interferon alpha beta receptor, *IL 17* Interleukin 17, *CD20* rituximab, *BLyS* Anti B lymphocyte Stimulator, *PDE 4* Phosphodiesterase 4, *RANKL* Receptor Activator of NF-κB Ligand, *IgE* Immunglobulin E, *C5* Complement 5, *IL 5* Interleukin 5, *IL 4R/13R* Interleukin 4R/13R

### How to screen for latent tuberculosis?

- Screening for LTBI includes a medical history, an IGRA test and a chest X‑ray: 100% agreement.
- For non-immunosuppressed persons and for planned, low-risk medication (see heat map orange), a chest X‑ray can be dispensed with: 100% agreement.
- IGRA findings must always be well documented: 100% agreement.
- A TST is to be considered for special situations: 100% agreement.

Screening for LTBI includes a detailed history of possible tuberculosis exposure and other risk factors (previous tuberculosis infection, diabetes, smoking status, alcohol consumption, drug consumption, malnutrition, chronic kidney disease, cancer, etc.). Furthermore, an IGRA test should indeed be carried out. However, by its very nature, immunosuppressed persons are susceptible to a certain degree of error by this method, which can lead to false negative or inconclusive results. In particular, the use of glucocorticoids can lead to a false negative result. Therefore, a chest X‑ray should always be included in immunocompromised patients to exclude tuberculosis. A high resolution CT (HRCT) is not absolutely necessary.

The result and date of IGRA testing should be well documented to provide clarity for future practitioners.

The TST is to be considered as an alternative option due to the influence of the *Bacille Calmette-Guérin* (BCG) vaccination and the need for two visits within 3 days.

### What is the correct preventive therapy for latent tuberculosis?

- The following treatment regimens are available for preventive tuberculosis therapy:
  - Rifampicin (RIF) for 4 months
  - Isoniazid (INH) for 9 months
  - Combined RIF + INH for 3 months: 100% agreement
- Comorbidities, comedications, patient’s expected adherence to therapy as well as the availability of proper medication must be considered in the selection process: 100% agreement
- Preventive therapy should be well documented to provide clarity for future practitioners: 100% agreement
- After 4 weeks of preventive tuberculosis therapy (at the earliest), treatment with a bDMARD or a tsDMARD can be started presupposing a satisfactory patient tolerance: 100% agreement

According to the 2018 WHO guidelines, the 2017 2SK guidelines and the National Tuberculosis Controllers Association and CDC guidelines of 2020, there are four treatment regimens to choose from for preventive tuberculosis therapy. Since rifapentine is not available in Austria and need not be taken weekly, this therapy option was eliminated. The three remaining therapy regimens appear in all three guidelines in a slightly different order of recommendation, thus it was decided to recommend all three therapy regimens equally. In our group discussion, regional differences in the application of the treatment regimens became apparent, although all are currently used in Austria.

Advantages and disadvantages of individual therapeutic regimens reflect the spectrum of side effects inherent in the individual drugs, which must be considered in the therapeutic decision together with patient comorbidities and comedications (see Table 2). However, duration of treatment and drug availability are additional factors that should influence the decision. When taking INH, the simultaneous administration of vitamin B6 (pyridoxine) can reduce the risk of neurological side effects. Attention should be paid to vitamin B6 substitution, especially during pregnancy and in the case of pre-existing vitamin B6 deficiency or polyneuropathy.

**Table 2:** List of the most important side effects, contraindication, and interactions of isoniazid and rifampicin

	Isoniazid (INH)	Rifampicin
*Side effects*	Peripheral neuropathy, hematologic reactions (agranulocyosis, anemia, thrombocytopenia), mental disturbances, nausea, epigastric disorders, pancretitis, hepatitis/hepatotoxicity, exanthema	Dizzyness, thrombocytopneia, flu-like-syndrom, gastrointestinal disturbances, hepatitis/hepatotoxicity, exanthama, temporary discoloration of skin and body fluids (orange)
*Contraindications*	Hypersensitivity, peripheral neuropathy, severe bleeding tendency, severe liver disease	Hypersensitivity, severe liver disease porphyria, simultanous use of Saquinavir/Ritonavir
*Drug interactions*	Barbiturates, phenytoin, carbamazepin, primidon. rifampicin, valproic acid, acetaminophen, ketoconazol, disulfiram, alcohol, antacids, levodopa	CYP450-inductor—multiple interactions, especially antiepileptics, benzodiacepine, paracetamol, azol-antifungal agents, antiviral therapies, antiarrhythmics, …

The importance of maintaining clear documentation protocols for any preventive therapy, including the drug and duration, was included as an important single point in the consensus to provide clarity for future medical practitioners to the benefit of their patients.

Table 3 shows the three treatment regimens and dosages.

**Table 3:** Treatment regimens for preventive therapy of latent tuberculosis infection (*LTBI*)

Treatment regimens for preventive therapy of LTBI	
Isoniazid (INH)	9 months
Rifampicin (RIF)	4 months
INH + RIF	3 months
*Dosage:*	
INH: 5 mg/kg, maximum 300 mg/day	
RIF: 10 mg/kg, maximum 600 mg/day	

### Which protocols should be followed during preventive therapy?

- Before commencing with preventive tuberculosis therapy, a medical history review, patient education and basic laboratory testing (blood count, alanin aminotransferase (ALT), aspartat aminotransferase (AST), gamma-glutamyltransferase (GGT), alcalic phosphatase (AP), bilirubin, creatinine) should be carried out: 100% agreement
- During preventive tuberculosis therapy, blood count, ALT, AST, AP, GGT, bilirubin and creatinine should be determined initially after 2 weeks, then every 4 weeks: 100% agreement
- In the case of pre-existing liver disease, individualized control intervals are carried out: 100% agreement
- Particular caution is advised when combining with potentially hepatotoxic drugs (methotrexate, leflunomide, azathioprine): 93% agreement
- In the case of a transaminase increase > 3 times the normal upper limit, weekly controls should be carried out; in the case of an increase > 5 times the normal upper limit, treatment should be discontinued: 100% agreement

There is only scarce evidence and no clear guidelines are available on this topic, so that within the consensus meeting, followed by a discussion with a hepatologist (HH), we found a pragmatic, yet safe procedure that earned the agreement of all participants. It was decided that a detailed anamnesis with regard to previous liver disease, alcohol consumption and comedication (potential interactions) should be taken beforehand. Furthermore, patients should be enlightened to the symptoms of potential liver damage (upper abdominal pain, icterus, dark urine, acholic stool, itching, anorexia, nausea) and matters regarding contact persons and any necessary measures should be discussed. Information about alcohol consumption and the use of additional medication (self-medication such as paracetamol) should also be provided.

The control intervals of laboratory tests were fixed at initially 2‑weekly then 4‑weekly. If the values increase, narrower intervals should be selected and are to be designed individually. Every new increase in liver values should also be evaluated with regard to intermediate liver diseases, which means more frequent testing intervals. In particular, if the transaminases rise above the threefold normal upper limit, weekly controls should be carried out. If the transaminases rise above 5 times the normal upper limit, treatment should be discontinued.

The highlighting of methotrexate in this context serves to draw the practitioner’s attention to this potentially hepatotoxic drug, which is frequently prescribed in rheumatologic patients. A treatment break during tuberculosis therapy should also be considered for methotrexate.

In addition, it was stated in a separate point that in the case of pre-existing liver disease, the control intervals specified here are not presented strictly as a guideline, but rather to encourage a more cautious approach.

### What to do if preventive therapy is not well tolerated?

- In cases of intolerance for one tuberculosis drug, the other available drug should be given: 100% agreement
- In cases of intolerance for both tuberculosis drugs, a low-risk anti-rheumatic drug should be substituted to support the basic rheumatological therapy. (see heat map green or yellow): 100% agreement
- In the absence of treatment for LTBI, prescribing a drug from the orange/red area of the heat map requires verbal and written informed consent by the patient after a thorough benefit-risk assessment along with close monitoring throughout: 93% agreement

If an intolerance to the first-line tuberculosis drug (INH, RIF) becomes apparent, it is possible to switch to a regimen with an alternative drug. However, if the feasibility of preventive therapy is not given due to an intolerance for both drugs, an alternative anti-rheumatic treatment with low risk of tuberculosis activation should be prescribed. Combinations of conventional DMARDs (cDMARDs) and bDMARD/tsDMARD with a low tuberculosis risk (heat map: green, yellow) can be used for this. However, if there are no alternative treatment options for the patient, the decision to intiate a bDMARD/csDMARD therapy prescribing a drug from the orange (red) area of the heat map can still be made—with detailed information about the benefits and risks of such a treatment shared with the patient. It is essential that this procedure is documented in writing. Subsequently, both sides must pay close attention to the occurrence of possible symptoms of a mycobacterial infection in order to enable detection at a very early stage.

### What is the best subsequent procedure upon completion of tuberculosis therapy?

- After a fully completed therapy for tuberculosis or latent tuberculosis, no further tuberculosis therapy needs to be given (even with a positive IGRA result) (except in the case of a proven new infection): 100% agreement

If a patient had a prior tuberculosis infection that was adequately treated, it can be assumed that all mycobacteria have been destroyed and no further preventive tuberculosis therapy is therefore necessary. A positive IGRA does not reflect a latent infection in this case but is a relict of the prior infection [45].

Similarly, complete elimination of mycobacteria can be expected after fully implemented preventive therapy, so that a continued positive IGRA can be ignored without evidence of a new infection.

### What to do if IGRA is not conclusive?

- If IGRA is repeatedly inconclusive, further testing (other IGRA, TST) would be indicated: 100% agreement
- If the test result is still inconclusive, a bDMARD or tsDMARD may be given without preventive tuberculosis treatment if there is no evidence of tuberculosis in the computed tomography (CT) of the lungs: 86% agreement

An inconclusive IGRA result may be caused by immunodeficiency, which can result from either a prior affliction or as a result of immunosuppressive therapy. Steroid therapy plays a major role here, therefore it is essential to perform the IGRA test at a time when no or at least only low amounts of glucocorticoids are being taken.

However, if the test result is repeatedly inconclusive, retesting should be attempted with an alternative product (IGRA of a different manufacture, TST).

If clear findings are still not achieved, a CT of the lungs can be used to exclude indications of an active or existing tuberculosis infection. If there is no clinical or CT evidence of tuberculosis infection, bDMARD/csDMARD treatment may commence without preventive tuberculosis therapy in a low tuberculosis incidence country such as Austria.

### When is it necessary to repeat the IGRA?

- Repeating a previously negative IGRA during ongoing treatment with a bDMARD/tsDMARD or when changing a bDMARD/tsDMARD, is only indicated in the event of clinical suspicion (e.g. contact with tuberculosis, travel to an endemic area): 93% agreement

During ongoing bDMARD/tsDMARD treatment, it is not routinely necessary to repeat IGRA. Switching from one bDMARD/tsDMARD to another does not necessarily require retesting.

Of course, testing can be done at any time, especially if there is a relevant risk of exposure. This includes stays in areas with a higher incidence of tuberculosis, contact with persons with tuberculosis or precarious living situations.

A summary of the consensus results is shown in Fig. 3.

**Fig. 3:**
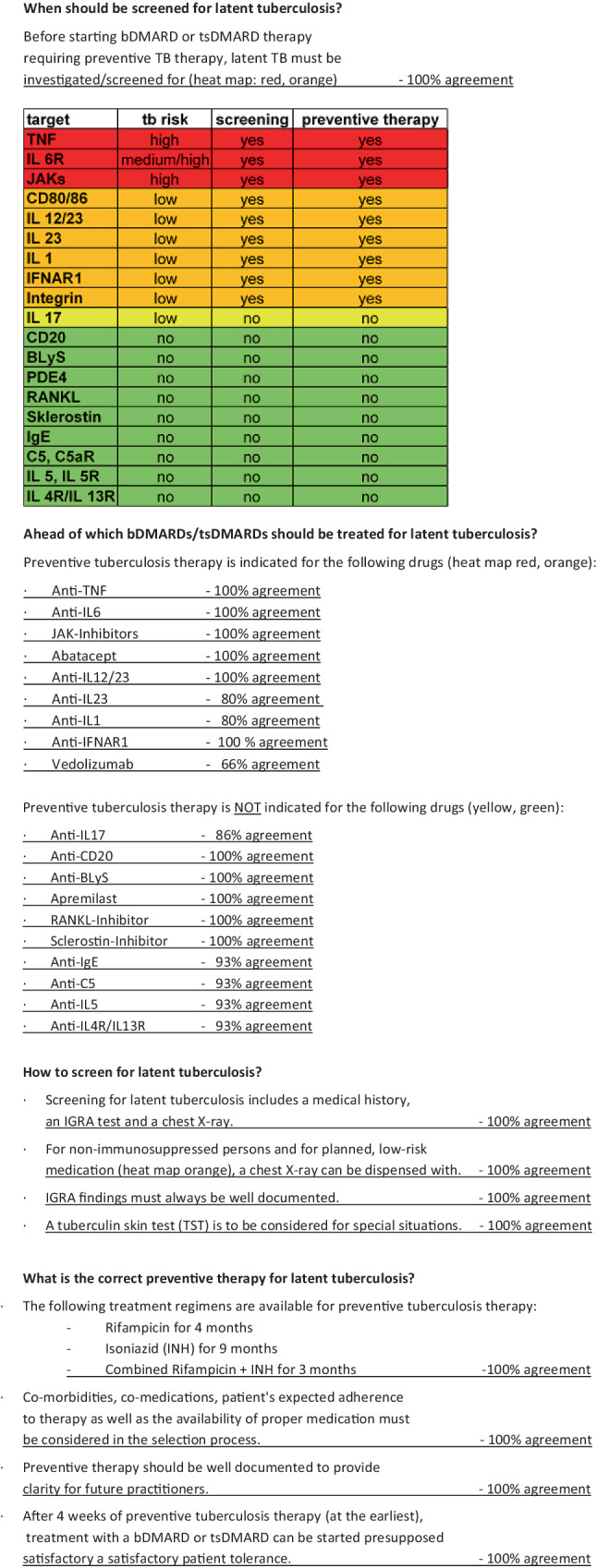
Consensus statements and percentage of agreement

**Fig. 3:**
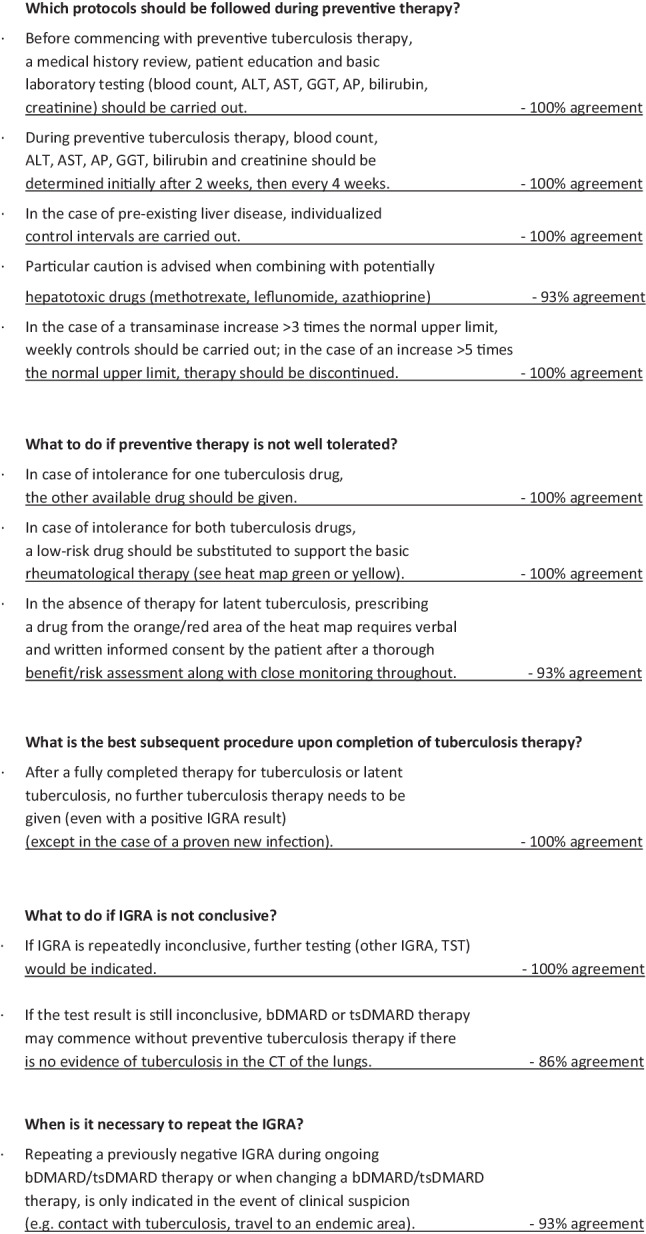
(continued)

## Discussion

Austria-wide standardization of the approach to use bDMARDs/tsDMARDs in patients with LTBI was a major factor in the preparation of this consensus statement. We are aware that not all questions can be answered with complete evidence, but experience with various therapies and the large number of available publications on most medications do nevertheless enable a very good assessment of the risk for tuberculosis infections. In addition, due to the fact that Austria is considered a low incidence country for tuberculosis, further risk reduction with regard to tuberculosis is apparent. This led to the classification of IL 17 blockers in the low-risk group and thus to the waiving of screening for LTBI. Other drug classes (heat map orange) would portray a similarly low risk of tuberculosis in the estimation of the participants, but screening and preventive therapy were deemed reasonable in light of the information included in the technical product summaries. Future controlled, randomized studies would likely increase certainty around this issue.

With regard to screening methods, this consensus should also lead to simplification and standardization of an accepted approach within the medical profession.

The selection and monitoring of preventive therapies, as set out in this consensus, also aims to facilitate their use and minimize potentially negative effects for patients.

Based on the statements listed here, handling bDMARDs/tsDMARDs should be less cumbersome for all physicians working in Austria and all relevant questions regarding LTBI should be further clarified based on currently available knowledge.
